# Green Synthesis of Controlled Shape Silver Nanostructures and Their Peroxidase, Catalytic Degradation, and Antibacterial Activity

**DOI:** 10.3390/jfb14060325

**Published:** 2023-06-18

**Authors:** Ayesha Shafiq, Aarti R. Deshmukh, Khaled AbouAitah, Beom-Soo Kim

**Affiliations:** Department of Chemical Engineering, Chungbuk National University, Cheongju 28644, Republic of Korea; engr.ayesha.shafiq@gmail.com (A.S.); aartideshmukh1@gmail.com (A.R.D.); ke.abouaitah@gmail.com (K.A.)

**Keywords:** green synthesis, silver nanodendrites, peroxidase activity, dye degradation, antibacterial activity

## Abstract

Nanoparticles with unique shapes have garnered significant interest due to their enhanced surface area-to-volume ratio, leading to improved potential compared to their spherical counterparts. The present study focuses on a biological approach to producing different silver nanostructures employing *Moringa oleifera* leaf extract. Phytoextract provides metabolites, serving as reducing and stabilizing agents in the reaction. Two different silver nanostructures, dendritic (AgNDs) and spherical (AgNPs), were successfully formed by adjusting the phytoextract concentration with and without copper ions in the reaction system, resulting in particle sizes of ~300 ± 30 nm (AgNDs) and ~100 ± 30 nm (AgNPs). These nanostructures were characterized by several techniques to ascertain their physicochemical properties; the surface was distinguished by functional groups related to polyphenols due to plant extract that led to critical controlling of the shape of nanoparticles. Nanostructures performance was assessed in terms of peroxidase-like activity, catalytic behavior for dye degradation, and antibacterial activity. Spectroscopic analysis revealed that AgNDs demonstrated significantly higher peroxidase activity compared to AgNPs when evaluated using chromogenic reagent 3,3′,5,5′-tetramethylbenzidine. Furthermore, AgNDs exhibited enhanced catalytic degradation activities, achieving degradation percentages of 92.2% and 91.0% for methyl orange and methylene blue dyes, respectively, compared to 66.6% and 58.0% for AgNPs. Additionally, AgNDs exhibited superior antibacterial properties against Gram-negative *E. coli* compared to Gram-positive *S. aureus*, as evidenced by the calculated zone of inhibition. These findings highlight the potential of the green synthesis method in generating novel nanoparticle morphologies, such as dendritic shape, compared with the traditionally synthesized spherical shape of silver nanostructures. The synthesis of such unique nanostructures holds promise for various applications and further investigations in diverse sectors, including chemical and biomedical fields.

## 1. Introduction

Recently, there has been a notable surge for nanoparticles in multiple applications and products that support human life. High-quality metal nanoparticles (e.g., Au, Ag, Pt, and others) have gained extensive research in diverse biomedical domains, e.g., anticancer therapy, radiotherapy augmentation, drug delivery, antibacterial treatments, diagnostic assays, antifungal treatments, bioimaging, biosensing, gene delivery, and numerous others [1,2,3]. When concerning silver nanoparticles, they are utilized in composites [4,5], ceramics, polymers, agriculture, and energy [6]. Several methods have been employed to synthesize nanoparticles, including the chemical process, physical process, biological process, thermal decomposition [7], heat-induced evaporation, and laser ablation [8]. Chemical methods typically involve reduction using reducing agents (i.e., sodium borohydride, dimethylformamide, trisodium citrate, hydrazine, ascorbic acid, m-hydroxy benzaldehyde, oleyl amine, and polyvinylpyrrolidone). Nanoparticles produced by these techniques range in particle size from 25 nm to 650 nm [9]. However, compared with the biological approach, the production cost and toxicity of hazardous chemicals are disadvantages. Biological methods typically utilize microorganisms such as algae, fungi, and bacteria that are safe as bio-reducing agents, but their synthesis rates are slower compared with green synthesis using plant-based materials. Therefore, among biological methods, green synthesis, which utilizes plant materials (biomass, juice, and extracts) to synthesize silver nanoparticles, is increasingly supported by researchers. This is due to simplicity, eco-friendliness, safety, cost-effectiveness, reproducibility, stability, and source availability [10].

In the current study, the *Moringa oleifera* plant, a member of the *Moringa* family commonly used as a vegetable in Asian countries, was employed as a bio-reducing agent. *Moringa oleifera* is a fast-growing, draft-resistant plant native to Asian countries such as Nepal, Pakistan, and India. It is also grown in tropical and subtropical regions of America and Africa [11] and is readily available in local markets for a variety of purposes. This plant exhibits a variety of benefits [12]. For example, from a nutritional perspective, *Moringa* leaves contain protein, zinc, potassium, magnesium, and copper [13]. It is rich in natural bioactive components with medicinal properties such as flavonoids, phenolic compounds, terpenoids, carotenoids, sterols, anthraquinones, alkaloids, and saponins [14,15,16,17]. Some flavonoids demonstrate anticancer activity against Hela cancer cells [18]. In the realm of nanoscience, *Moringa oleifera* has received huge attention over the past decades for its potential ability to synthesize various metal nanoplatforms, as reported in various studies related to iron oxide, nickel oxide, lanthanum oxide [19], magnesium oxide [20], tungsten [21], and palladium nanoparticles [22] and others [23].

Important requirements in the development of nanoparticles are size and shape, as crucial factors that determine the reactivity and use of nanoparticles. Thus, changes in these properties can have a significant impact on their applications [24]. It is acknowledged that nanoparticles with complex structures have received more attention than simple particles due to their intricate structure [25]. Particle morphology, such as silver nanoflowers, silver dendrites, silver nanostars, and silver nanowires, provides more surface area or reactive sites compared with spherical nanoparticles [26]. Several studies have been conducted using trisodium citrate [27], ammonium citrate dibasic [28], polyvinylpyrrolidone, and formamide [29] to achieve nanoflower-like morphology.

The current study provides a straightforward method for the green synthesis of silver nanostructures in a controlled shape using *Moringa oleifera* leaf extract. Interestingly, two distinct morphologies of silver nanostructures, dendritic shapes (AgNDs) and spherical shapes (AgNPs), were feasibly obtained for the first time by varying the concentration of plant extract as a critical factor when reacted with silver nitrate with and without copper ions in the reaction mixture. Our results showed that silver nanodendrites exhibit superior properties compared with spherical nanoparticles when evaluated for oxidation, catalytic degradation, and antibacterial activity. These findings could open many opportunities and possible applications in various fields.

## 2. Materials and Methods

### 2.1. Synthesis of Silver Nanoparticles

Chemicals in all studies were used as purchased. Distilled water used in the experiment was produced with a Green RO 350 water purification system (Seoul, Korea). *Moringa oleifera* dried leaves were purchased from the Hands Herb Company, Korea. Leaves were rinsed with plenty of distilled water before drying again at 80 °C using an oven. The dried leaves were then ground with a mortar and pestle and stored in a glass bottle until use. For plant extraction, 3 g of dried leaves were added to 100 mL distilled water and refluxed at 100 °C for 1 h. Next, the solution was filtered and stored at 4 °C. To synthesize silver nanostructures, 5 mM AgNO_3_ (silver nitrate, Samchun Chemicals Company, Seoul, Korea) and 3 mM of Cu (NO_3_)_2_ · xH_2_O (copper nitrate (II) hydrate 99.99%, Sigma-Aldrich, St. Louis, MO, USA) were added together to a 20 mL distilled water solution. Plant extracts of different volumes were added dropwise to this solution to form silver nanoparticles with a controlled shape effectively. Two types of experiments were performed to determine the growth mechanism. The first type of experiment involved fixing precursor concentration (metal salt) and varying the volumetric concentration of the reducing agent (plant extract) as 10%, 20%, 30%, and 40% of the precursor, while the second type involved fixing the concentration of plant extract and varying the volumetric concentration of metal salt as 12.5%, 16.6%, and 25% of the precursor. These experiments were performed with and without copper nitrate hydrate. The mixture solution of each designed sample was incubated at room temperature for 24 h with a stirring speed of 200 rpm and then centrifuged (15,000 rpm for 10 min) using a Hanil Mega 17r high-speed refrigerated centrifuge, Korea. After removing the supernatant, the pellet precipitate was washed several times with distilled water. Finally, nanoparticles were obtained using a freeze dryer (Ilshin, Korea).

### 2.2. Peroxidase Activity

Peroxidase assay was performed according to Deshmukh and colleagues [30]. Briefly, 0.15 mL of concentrated AgNDs/AgNPs suspension (50 μg/mL) was mixed with 0.1 mL (10 mM) hydrogen peroxide (H_2_O_2_) (Samchun Chemicals Company, Seoul, Korea) in a test tube and allowed to stand at room temperature. Then, 0.25 mL acetate buffer solution (pH 4.2) was added, followed by rapid addition of 0.2 mL (12 mM) of 3,3′,5,5′-tetramethylbenzidine (TMB, 99%, Sigma-Aldrich, St. Louis, MO, USA) in ethanol. The colorless TMB was oxidized to a blue diimine. Absorbance was monitored over time, and comparisons were recorded at 655 nm at regular intervals [31].

### 2.3. Catalytic Activity

Two types of dyes were investigated to evaluate the catalytic behavior of silver nanoparticles in degradation. Methyl orange (MO, 85% dye content), methylene blue trihydrate (MB, 97% dye content), and sodium borohydride (NaBH_4_, extra pure) were purchased from Samchun Chemicals Company, Seoul, Korea. A catalytic dye degradation experiment was performed by adding 5 mL of 15 mg/L dyes (MO, MB) to a 15 mL test tube, followed by adding 50 μL (0.06 M) NaBH_4_ to each tube. The catalytic behavior was observed by adding 150 μL of 100 μg/mL AgNDs or AgNPs to this solution. The reaction started immediately, and the solids were removed after 5 min by centrifugation (13,000 rpm for 10 min). The degradation percentage was measured by recording the optical density using a UV–Vis spectrophotometer.

The % dye degradation was calculated from the equation:

(1) $$\%\;\mathrm{Degradation}=\frac{\left(c_{0}-c_{t}\right)}{c_{0}}\times 100$$

where C_0_ represents the initial dye concentration, and C_t_ represents the final dye concentration after degradation.

### 2.4. Antibacterial Activity

The antibacterial activity of AgNDs and AgNPs was evaluated using the disk-diffusion agar method [32] against representative microorganisms, *Staphylococcus aureus* (ATCC 6538) and *Escherichia coli* (ATCC 11775). Bacteria were cultivated on Luria-Bertani (LB) medium and incubated at 37 °C for 24 h. Antibacterial tests were performed using nanoparticle concentrations (100 μg/mL) [33,34] as effective concentrations against bacterial strains. After dropping 100 μL of silver nanostructures (100 μg/mL) on a sterile paper disc (Advantech, Japan) with a diameter of 10 mm, it was dried, placed on an LB agar plate, and incubated at 37 °C for 24 h. Then, the antibacterial activity was obtained by measuring the zone of inhibition diameter (mm). Antibacterial activity was assessed in triplicate, and data were plotted with error bars after calculating standard deviations.

### 2.5. Characterization of Silver Nanostructures

A UV–Vis absorbance spectrum of 200–800 nm wavelength was acquired using a UV–Vis spectrophotometer at a resolution of 10 nm (mini, Shimadzu, Japan). Fourier transform infrared spectroscopy (FTIR) analysis was used to identify functional groups. FTIR measurements were performed using an IR200 FTIR spectrometer (Thermo Scientific) with a wavenumber range of 500–4000 cm^−1^ and a resolution of 4 cm^−1^ after 32 scans. The crystallinity of the nanostructures was measured using an X-ray diffractometer (XRD, X’Pert-Pro, Analytical) under the following conditions: room temperature, voltage 40 kV, CuKα radiation at λ = 1.5406 Å, and scanning with 2θ range from 0° to 100° with 2°/min. The average crystallite size was determined by the Debye equation.

(2) $$D=\frac{0.9\;\lambda }{\beta \;\cos\;\theta }$$

where D is the particle size, λ is the X-ray wavelength (0.154 nm), and β is the full width at half maximum (FWHM). The interplanar spacing (d) between atoms was calculated by Bragg’s law.

(3) 2d sin θ=nλ

(4) $$d=\frac{\lambda }{2\sin\theta }\left(n=1\right)$$

The nanostructures morphology was characterized using transmission electron microscopy (TEM, Libra 120, Carl Zeiss, Jena, Germany). For TEM observation, drops of the colloidal nanostructure solution were placed on a carbon-coated copper grid and dried at room temperature. Particle morphology and elemental composition were analyzed using a scanning electron microscope (SEM, LEO-1530) coupled to energy-dispersive X-ray spectroscopy (EDS). X-ray photoelectron spectroscopy (PHI Quentera-II) analysis was performed to investigate the chemical oxidation state and surface composition, providing compositional information at the top of the monolayers, with a detection limit of 0.01–0.5 atomic%, an analysis depth of 0.5–7.5 nm, and a resolution of ≤10 μm probe size.

## 3. Results and Discussion

Silver nanostructures were synthesized utilizing *Moringa oleifera* extract from leaves due to its properties as a reducing and capping agent. A green synthesis approach to metal nanoparticles can incorporate carbohydrates, amino acids, proteins, phenolic compounds, and flavonoids that promote metal ions to reduce and stabilize nanostructures [35,36].

### 3.1. Microscopic Observation of Silver Nanostructures

**Figure 1:**
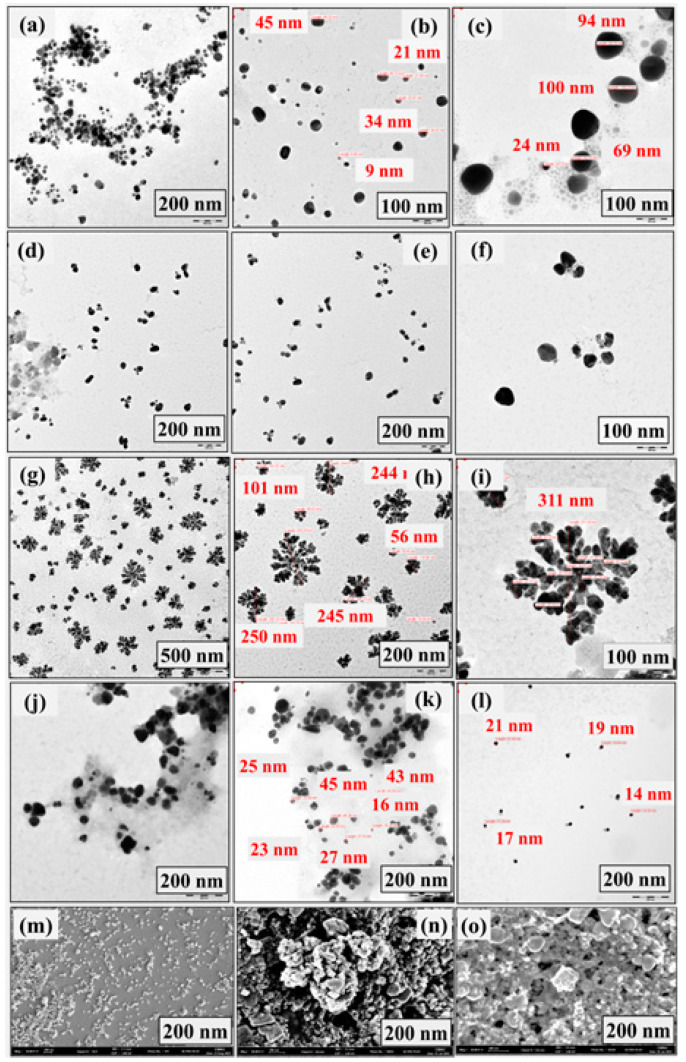
Morphologies of synthesized silver nanostructures by TEM and SEM observations. (**a**–**c**) TEM images of silver nanospheres of different sizes in the presence of Cu^2+^ ions. (**d**–**f**) Deformed TEM images of spheres for particle bud formation in the presence of Cu^2+^ ions. (**g**–**i**) Stabilized silver nanodendrites in the presence of Cu^2+^ ions. (**j**–**l**) AgNPs in the absence of Cu^2+^ ions. (**m**) SEM image of silver nanospheres. (**n**,**o**) SEM images of silver nanodendrites.

The obtained results reveal different morphologies depending on the plant extract concentration in the presence and absence of Cu^2+^ ions. The nanostructures obtained at a low concentration of 10% of the precursor with Cu^2+^ ions in the reaction system are spherical with a size of about 100 ± 30 nm, as shown in Figure 1a–c. Increasing the plant extract concentration to 20% in the presence of Cu^2+^ ions results in a transition state that slightly changes the spherical shape to an unstable and undefine structure, as shown in Figure 1d–f. Afterward, the plant extract concentration was increased to 30% to investigate the possibility of changing the shape of the nanostructure. It is found that the shape of dendrites is clearly formed as the concentration of the plant extract increases (Figure 1g–i). The nanodendrites have an average size of ~300 ± 30 nm. Figure 1j shows the results obtained in the absence of copper nitrate hydrate when using a low concentration of 10% of precursor. The obtained nanoparticles are spherical at this concentration, and no dendritic morphology was observed even when the concentration was increased to 30% of the precursor (as shown in Figure 1k,l). Therefore, for the formulation of nanodendrites, the volumetric concentration of plant extract should be as high as 30% in the presence of Cu^2+^ ions. Additionally, Figure 1m shows the SEM observation results for AgNPs, and Figure 1n,o displays AgNDs.

The phytochemicals in the extract act as a reducing agent for the silver ions. One possible mechanism for the reduction of silver nitrate by phytoextract can be hypothesized as the following: Initially, silver nitrate exists as Ag^+^ and NO^3−^ ions in solution. The bioactive molecules in the plant extract provide hydroxyl and carbonyl groups donating electrons to silver ions, causing bio-reduction of Ag^+^ to Ag^0^. These reduced silver ions start to aggregate and form clusters. These clusters further grow and eventually stabilize to form silver nanoparticles. Consistent with this hypothesis, many studies have disclosed that flavonoids from various plant extracts exhibit bio-reductive functions of metal ions through a mechanism of keto-enol tautomeric transformation [37,38].

The above results show that AgNPs and AgNDs can be successfully prepared using *Moringa oleifera* leaf extract in the presence of Cu^2+^ ions. Cu^2+^ ions are necessary for nanodendritic formulation. The addition of Cu^2+^ ions can slow down the reaction, forming thermodynamically unstable branched structures that provide high specific surface areas. These branches then aggregate to reduce the uncovered area and create a self-assembled, stabilized dendritic structure [39,40]. Importantly, the shape of the nanoparticles in the synthesis system changes from spherical to dendritic as the concentration of the plant extract increases with the addition of Cu^2+^ ions. Increasing the concentration of the metal salt was found to have no effect on the morphology. Without Cu^2+^ ions in the system, there was no change in morphology, even at high plant extract concentration (30%). Therefore, for the formulation of nanodendrites, the volumetric concentration of plant extract should be as high as 30% in the presence of Cu^2+^ ions. Xu et al. [41] reported similar observations for the chemically synthesized 3-dimensional silver nanostructures in the presence of Cu^2+^ ions.

### 3.2. UV–Vis Spectroscopy and Elemental Analysis

**Figure 2:**
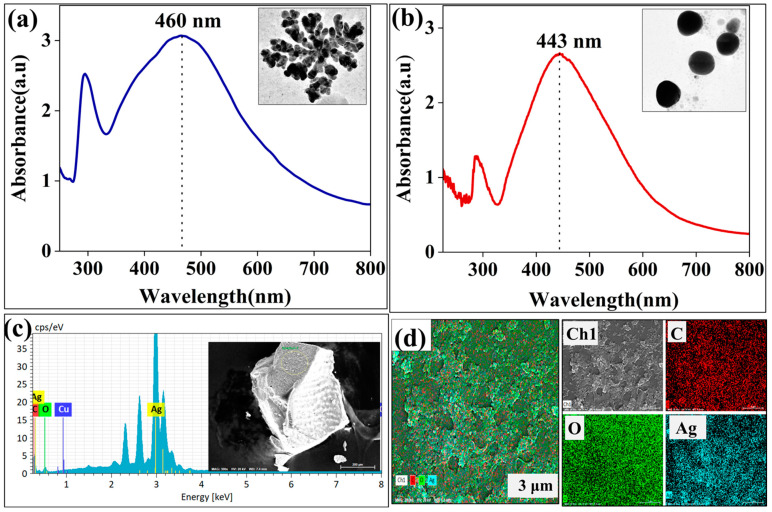
UV–Vis spectra and EDS elemental analysis of silver nanostructures. (**a**) UV–Vis spectra of silver nanodendrites. (**b**) UV–Vis spectra of silver nanospheres. (**c**) EDS spectrum of silver nanodendrites with the selected region. (**d**) EDS mapping of silver nanodendrites.

Figure 2 demonstrates possible differences in the optical properties of nanomaterials depending on the shape and size formed. According to the results, AgNDs show a broader spectrum with a maximum of 460 nm (Figure 2a), while AgNPs exhibit a narrower band with a maximum of 443 nm (Figure 2b). The obtained data is like previous data showing different morphologies for silver nanoparticles [42]. The elemental content of AgNDs was analyzed. Figure 2c clearly reveals that AgNDs have a strong absorption peak (3 keV), which is typical for silver nanocrystals [43]. Mass percentage analysis indicates that silver is predominantly present at 88.4%, along with the organic content on the surface of the silver nanodendrites, especially C (4.69%) and O (6.41%), while the Cu content is negligible at 0.72%. It has been reported that *Moringa oleifera* cannot induce copper nanoparticles under room temperature conditions and that high temperatures (≥80 °C) are required for the formation of copper nanoparticles [44], but high temperatures were not used in our conditions. This indicates that copper nitrate does not act as a strong competitor but slows down the reaction rate for silver nanoparticles formation. Figure 2d also presents the EDS mapping demonstrating the Ag atoms in AgNDs and confirming the distribution of the major elements in the nanoparticles.

### 3.3. FTIR Analysis

**Figure 3:**
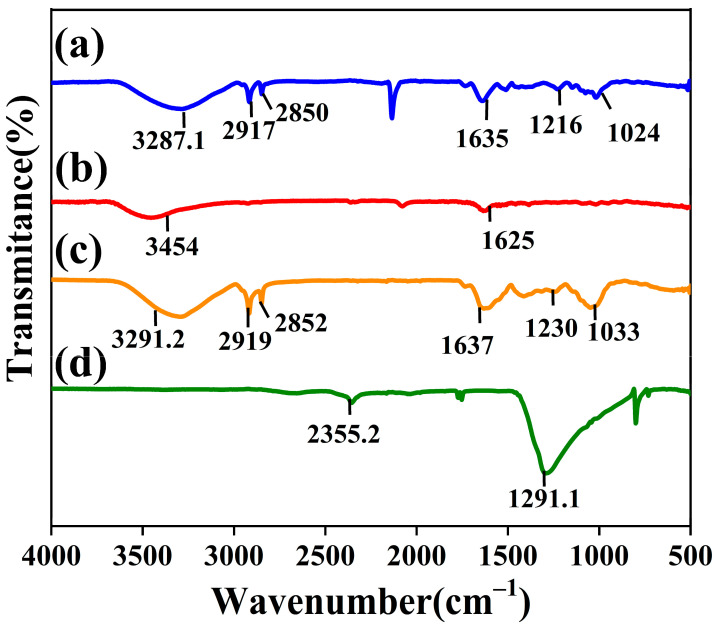
FTIR spectrum of (**a**) silver nanodendrites, (**b**) silver nanospheres, (**c**) *Moringa oleifera* leaf powder, and (**d**) silver nitrate.

FTIR investigations were employed to determine whether bioactive compounds are found on the surface of nanoparticles. Figure 3a demonstrates that AgNDs exhibit a peak at 3287 cm^−1^ related to O–H stretching vibrations of hydroxyl groups present mainly in plant phenols and alcohols. Two peaks are observed at 2917 cm^−1^ and 2850 cm^−1^, which are associated with C–H stretching vibrations. The peak observed at 1635 cm^−1^ can be attributed to C=C stretching (in aromatic rings present in terpenoids) and C=O ketones (in flavonoids). C-N bending vibrations appear at 1216 cm^−1^ and 1024 cm^−1^ related to the amide group of plant extract [45]. Figure 3b illustrates the FTIR spectrum of AgNPs. The broader spectrum detected at 3454 cm^−1^ can be ascribed to the O–H stretching. The peak seen at 1625 cm^−1^ is due to C=C and/or C=O stretching vibrations. Figure 3c displays the functional groups of *Moringa oleifera* extract leaf powder. The peak at 3291.2 cm^−1^ can be represented by the O–H stretching vibration, and the other peaks (2919 cm^−1^ and 2858 cm^−1^) can be confirmed by the C–H stretching of the alkane group. The peak at 1637 cm^−1^ can be assigned to C=C stretching and C=O. The peaks seen at 1230 cm^−1^ and 1033 cm^−1^ are associated with C–N bonds in the amine group [46]. From the FTIR results, it is predicted that the C=O group is dominantly involved in the reduction, while C–H and C–N stretching are likely involved in the shaping of the nanoparticles because they are not observed in the silver nanosphere vibration analysis. These results for *Moringa oleifera* are like previously reported results [47]. The presence of identical peaks with a slight peak shift in nanoparticles and *Moringa oleifera* powder suggests that the synthesized nanoparticles are capped with bioactive functional groups present in this plant. Silver nitrate shows an intense peak at 1291.1 cm^−1^ [48], indicating an Ag^+^NO^3−^ ion pair, as shown in Figure 3d.

### 3.4. XRD Analysis

**Figure 4:**
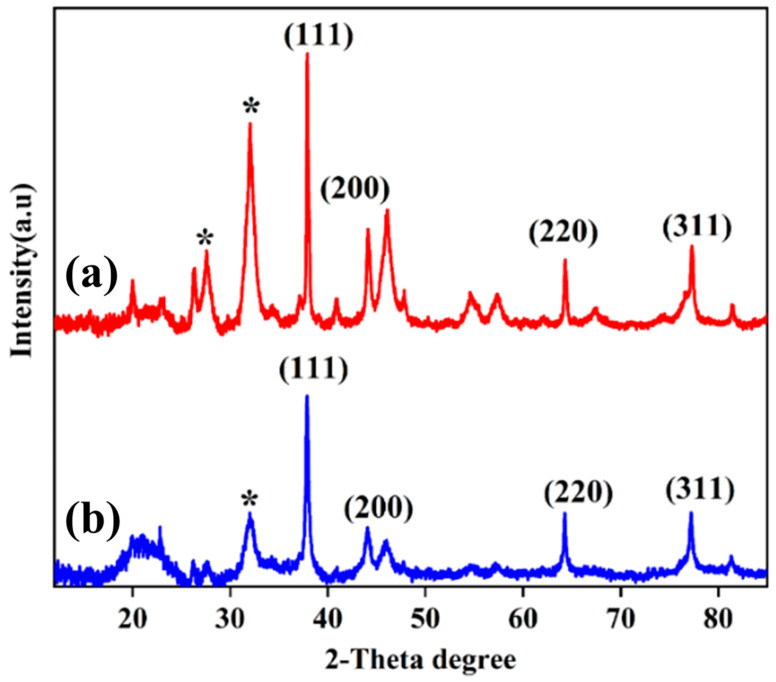
XRD analysis of (**a**) silver nanodendrites and (**b**) silver nanospheres. Extra peaks with (*) sign indicate the presence of organic compounds from *Moringa oleifera*.

Figure 4 indicates that both AgNDs and AgNPs have cubic face-centered (FCC) structures. As shown in Figure 4a, significant peaks and crystal planes are observed at 37.91° (111), 44.08° (200), 64.3° (220), and 77.30° (311) corresponding to d-spacings of 0.237 nm, 0.220 nm, 0.144 nm, and 0.123 nm for AgNDs. XRD analysis shows that the synthesized silver nanostructures correspond to well-defined surfaces with an arrangement of atoms in a crystal lattice manner. The intense peak at 2θ = 37.91° corresponds to the (111) reflection as one of the most compressed packed planes, indicative of the FCC structure. These results are in accordance with the standard powder diffraction card of a joint committee on powder diffraction standards (JCPDS), silver file No. 04-0783 [49]. In Figure 4b, distinct peaks and crystal planes are detected at 37.88° (111), 44.18° (200), 64.258° (220), and 77.203° (311) for AgNPs, which are attributed to d-spacings of 0.237 nm, 0.204 nm, 0.144 nm, and 0.123 nm. The approximate crystal size corresponding to (111) is 26.82 nm and 32.48 nm for AgNPs and AgNDs, respectively. The XRD data also reveals peaks representing organic compounds from the plant extract (32.104°, 37.16°, and 44.18°) in addition to peaks representing silver nanocrystals. These results are consistent with previous studies as XRD contains similar 2θ patterns [50,51]. Furthermore, no remarkable peaks are observed for the copper crystal due to its low presence in the reaction system.

### 3.5. XPS Analysis

**Figure 5:**
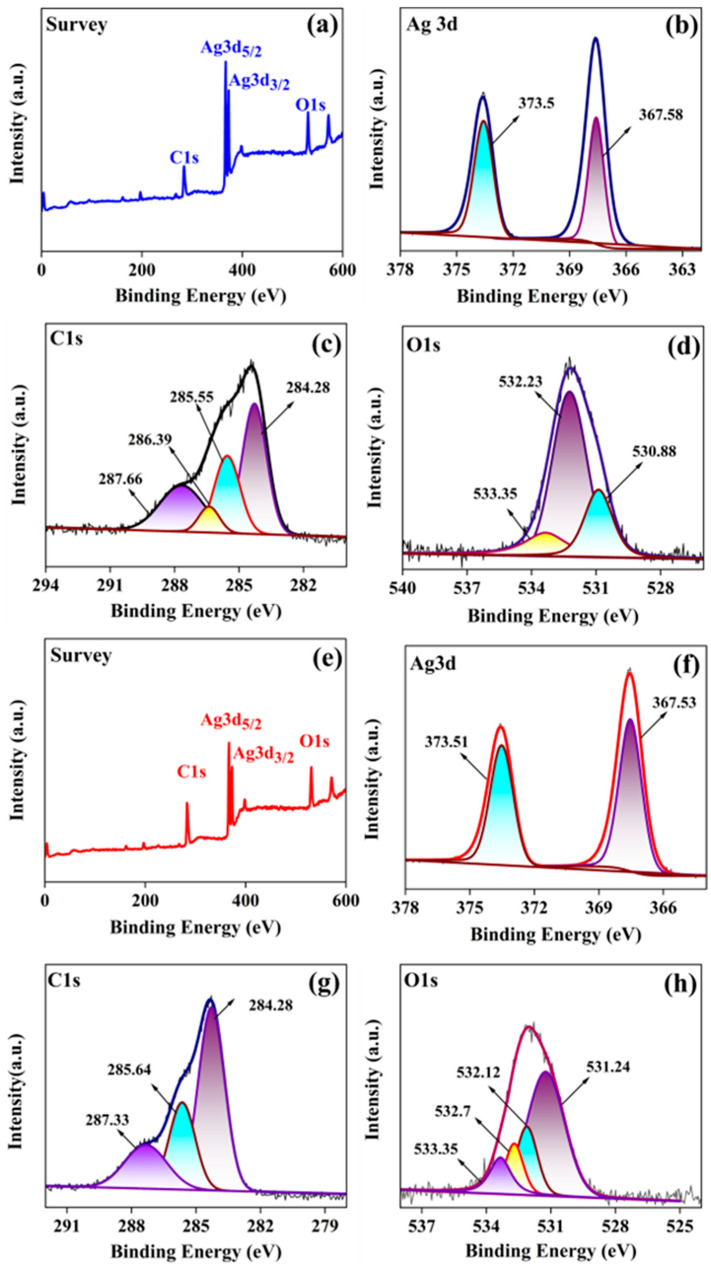
High-resolution XPS spectra of silver nanostructures. (**a**) Survey spectra of AgNDs. (**b**) Ag3d spectra of AgNDs. (**c**) Deconvoluted C1s spectra of AgNDs. (**d**) Deconvoluted O1s spectra of AgNDs. (**e**) Survey spectra of silver nanospheres. (**f**) Deconvoluted Ag3d spectra of AgNPs. (**g**) C1s spectra of AgNPs. (**h**) O1s spectra of AgNPs.

The synthesized silver nanostructures are further analyzed using XPS, as manifested in Figure 5. Figure 5a,e shows the survey spectra for AgNDs and AgNPs, confirming the presence of Ag^0^ as a major constituent. Ag peaks are detected at 365–376 eV (Figure 5b,f). As a spin-orbital splitting, the Ag3d peak is shown as a doublet with two states: 367.5 eV Ag3d_5/2_ and 373.5 eV Ag3d_3/2_ with splitting energy of 6 eV, confirming the state in Ag^0^ [52,53]. Figure 5c describes the deconvolution of C1s spectra from AgNDs with four signals at 284.28, 285.5, 286.39, and 287.66 eV with respect to the C=C, C–O, C–N, and C=O relationships [54,55,56]. The O1s XPS spectrum exhibits three signals at 530.88, 532.23, and 533.35 eV (Figure 5d) due to AgO, O–H, and C–O, respectively [57,58]. For AgNPs, the pattern gives a C1s spectrum (Figure 5g) with deconvolution into three peaks at 284.2, 285.6, and 287.3 eV, indicating the presence of C=C, C–O, and C–C connections [59,60,61]. The O1s XPS (Figure 5h) represents four peaks at 531.24, 532.12, 532.7, and 533.35 eV corresponding to the O–H, C=O, C–O linkages [62,63]. These results confirm the successful synthesis of silver nanostructures with *Moringa oleifera* leaf extract. Results are in accordance with FTIR studies confirming the connection.

### 3.6. Peroxidase Activity

**Figure 6:**
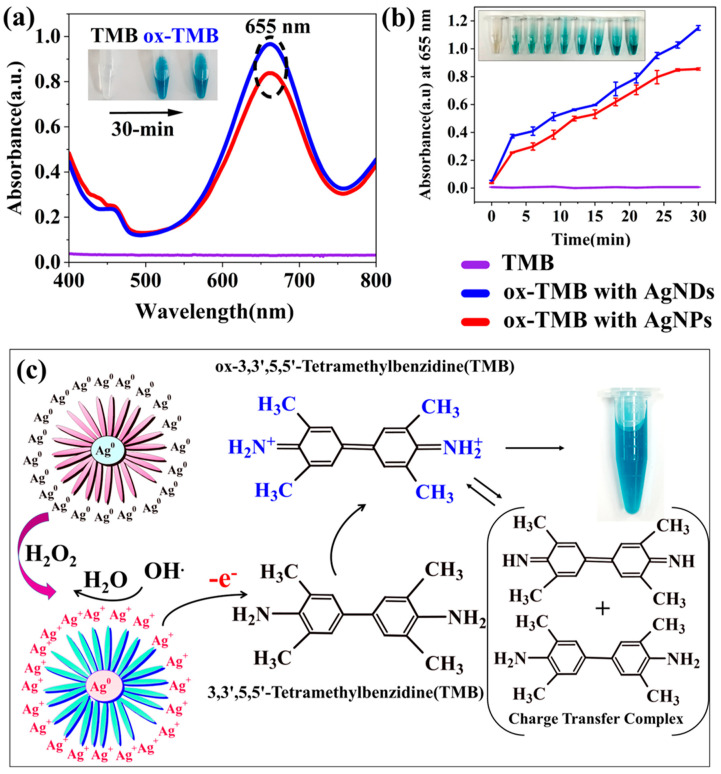
Oxidation of TMB in the presence of H_2_O_2_ on the surface of silver nanostructures. (**a**) Absorbance spectra of TMB, TMB + AgNPs, and TMB + AgNDs. (**b**) Changes in optical density at 652 nm at regular time intervals for TMB, TMB + AgNPs, and TMB +AgNDs. (**c**) Possible mechanisms related to the oxidation of TMB substrate in the presence of H_2_O_2_ facilitated by silver nanostructures.

Several nanoparticles are known to exhibit peroxidase activity, including cobalt tertraoxide [64], copper [65], nickel [66], gold [67], and silver nanoparticles [68]. In this study, the peroxidase activity of silver nanostructures was investigated using a chromogenic reagent TMB as shown in Figure 6. Originally, TMB did not exhibit any peak and appeared colorless, but when oxidation begins, with the release of Ag^+^ ions, colorless TMB undergoes oxidation and turns into a blue-colored diimine. The phenomena can be observed visually, and spectrometry analysis confirms the reaction as a band at 655 nm (Figure 6a). The absorbance of the samples was monitored over time, and comparisons were recorded specifically at 655 nm every 30 min with a spectral range of 400 to 800 nm, which gradually increases with rising TMB-oxide, as shown in Figure 6b. In contrast to AgNPs with a maximum OD of 0.8, AgNDs displayed higher oxidation, i.e., a maximum OD of 1.1. The plausible mechanism that could take place during this reaction and result in peroxidase activity is shown in Figure 6c. The process involves the following main steps: catalytic decomposition of H_2_O_2_ upon the addition of nanoparticles, and TMB oxidation via Ag^+^ ions, which is confirmed by a color change from colorless to blue. Initially, with H_2_O_2_, Ag^0^ undergoes Ag^+^ ion formation, which leads to the decomposition of H_2_O_2_ into OH radicals [69]. The free radicals then oxidize the peroxide substrate TMB, which confirms the formation of silver cation (Ag^+^) on the surface of silver nanostructures. In addition, the oxidized form of TMB can exist in equilibrium with the charge transfer complex and the diimine derivative for TMB.

### 3.7. Catalytic Degradation

**Figure 7:**
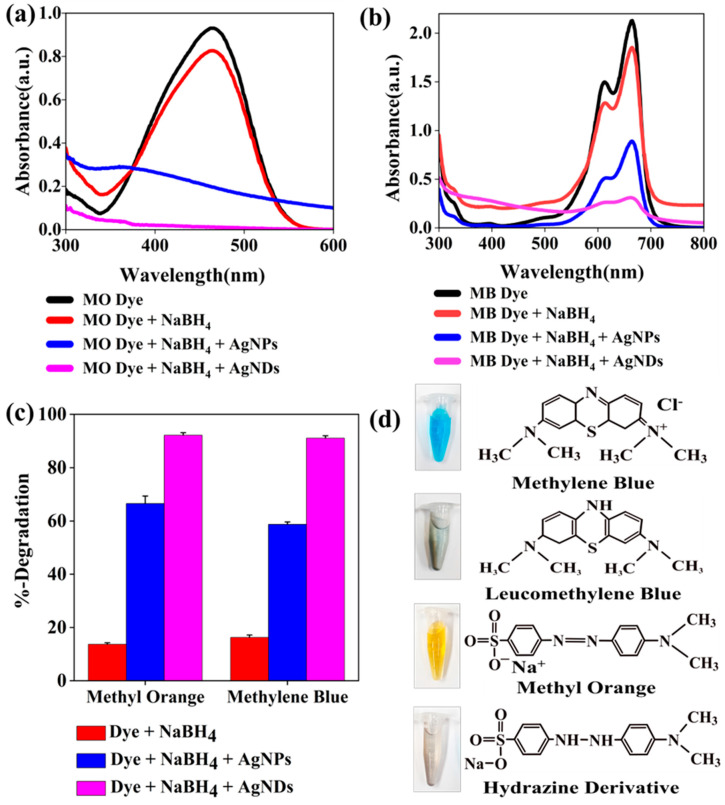
Catalytic degradation of dyes in the presence of NaBH_4_ on the surface of silver nanostructures. (**a**) Absorbance spectra of methyl orange, methyl orange + NaBH_4_, methyl orange + NaBH_4_ + AgNPs, and methyl orange + NaBH_4_ + AgNDs. (**b**) Absorbance spectra of methylene blue, methylene blue + NaBH_4_, methylene blue + NaBH_4_ + AgNPs, and methylene blue + NaBH_4_ + AgNDs. (**c**) Degradation percentage of dye + NaBH_4_, dye + NaBH_4_ + AgNPs, and dye + NaBH_4_ + AgNDs. Error bars show the standard deviation for three replicates of data. (**d**) Dye structures before and after reduction.

Methylene blue (MB) is a heterocyclic aromatic cationic dye that poses a threat to aquatic life due to its non-degradability and potential to cause carcinogenesis and toxicity. This dye is water-soluble and shows a dark blue color when dissolved in water and generates a significant signal at 664 nm when analyzed by a UV–Vis spectrophotometer. MB degrades to leuco-methylene blue [70]. Methyl orange (MO) is an anionic azo dye that displays a strong peak at 464 nm and is also water-soluble. The color ranges from orange, red to yellow depending on the acidity and alkalinity of the medium. When degraded, it forms hydrazine-derivatives [71]. Our results demonstrate the positive impact of silver nanostructures on the catalytic degradation of the used dyes (MO and MB). This is clearly shown in Figure 7a,b. Degradation of the dyes was slow when sodium borohydride was introduced, as observed both visually and by absorbance measurements. However, with the addition of silver nanostructures in the solution, the reaction rate was accelerated, and the reaction was completed in 3 to 5 min. The color of each dye disappeared, but black pellets were formed along with the colorless supernatant. In addition, the absorbance spectrum of the dyes was significantly reduced. These observations suggest that the degradation of dyes with sodium borohydride is thermodynamically favorable but not kinetically [72,73]. In this system, silver nanostructures act as a catalyst that increases the reduction/oxidation reaction of dyes with sodium borohydride [74,75].

The calculated degradation activity results show that AgNDs exhibit a higher degradation percentage compared to AgNPs (Figure 7c) as follows: 66.6% (AgNPs) and 92.2% (AgNDs) for MO dye degradation; 58.3% (AgNPs) and 91.0% (AgNDs) for MB dye degradation. The experiment was performed in triplicate, and the calculated standard deviation was less than 2%. It indicates that AgNDs have a superior catalytic effect required for the degradation of dyes.

A reasonable mechanism for this reaction to occur can be attributed to the rapid transfer of electrons available on the silver nanoparticles. Degradation activity relies on the doner NaBH_4_ capability and acceptor dye. Initially, dye molecules and BH_4_^–^ are adsorbed on the surface of nanoparticles [76]. Here, NaBH_4_ acts as a nucleophile, the dye molecules act as an electrophile, and the silver nanoparticles act as an electron relay system mixture that supports both electron transfer and degradation rate acceleration. These observations reflect the role of nanoparticles as substrates due to their high specific surface area [77,78]. For silver nanoparticles, several studies have demonstrated enhanced catalytic behavior for dye degradation with NaBH_4_ [79,80,81,82].

### 3.8. Antibacterial Activity

**Figure 8:**
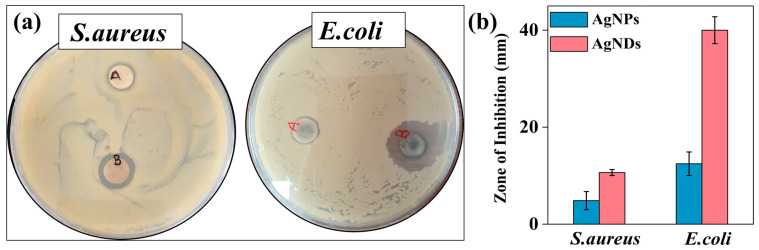
Antibacterial activity of nanostructures against Gram-positive and Gram-negative bacteria. (**a**) Antimicrobial activity of silver nanospheres (A) and silver nanodendrites (B) against *S. aureus* and *E. coli*. (**b**) Zone of inhibition (mm) of silver nanospheres (AgNPs) and silver nanodendrites (AgNDs) against representative bacteria. Error bars represent the standard deviation of triplicate experiments.

Silver nanostructures are primarily known for their potent antibacterial potential [83,84,85]. The antibacterial activity of the synthesized silver nanostructures was investigated against Gram-positive bacteria (*S. aureus*) and Gram-negative bacteria (*E. coli*). Figure 8a,b demonstrates the efficient killing of bacteria by AgNDs compared with AgNPs, showing specific antibacterial action when tested by the zone of inhibition. AgNDs have a higher antibacterial effect against *E. coli*, reaching an inhibition of 40 ± 1.5 mm, when compared with *S. aureus*, which had an inhibition of 10 ± 0.6 mm. In contrast, AgNPs treatment for both strains resulted in insignificant inhibition of 12.6 ± 1.2 mm against *E. coli* and 5 ± 0.91 mm against *S. aureus*. These results illustrate that nanoparticle shape enables antibacterial effectiveness depending on the morphology change. The possible antibacterial action mechanism of silver nanoparticles is explained as follows. Bacterial cells are composed of various structures, such as cell membranes, proteins, and DNA, which contain sulfur and phosphorus. They act as Lewis bases, while silver is considered a Lewis acid, resulting in an electrostatic attraction between sulfur proteins and silver ions [86,87]. Therefore, AgNDs can bind to the wall and penetrate bacterial cells [88,89]. Internalization of silver nanostructures into cells interrupts respiratory function, resulting in the deactivation of respiratory enzymes with the generation of reactive oxygen species (ROS) [90,91]. As such, overproduction of ROS can destroy intercellular components such as DNA, lipids, and proteins. Cellular membrane destruction thus causes loss of cytoplasm from the cell, followed by cell death. Additionally, cell wall thickness can determine the antibacterial efficiency of different bacteria upon contact with silver nanoparticles. Gram-negative *E. coli* has a thinner cell wall, making it more susceptible to silver nanoparticle penetration than Gram-positive bacteria such as *S. aureus*, which are characterized by thicker cell walls [92]. The structural shape of silver nanoparticles is also important in determining antimicrobial efficacy, and the dendritic shape possesses a more reactive crystal surface than the spherical-shaped nanoparticles, leading to enhanced antibacterial performance [93,94,95].

## 4. Conclusions

In search of an environmentally friendly method to produce nanostructures, a green synthetic route was introduced to obtain controlled silver nanostructures, dendrites (AgNDs), and spheres (AgNPs) using *Moringa oleifera* leaf extract at room temperature. These nanostructures were characterized by surfaces containing bioactive functional groups. Dendritic silver nanostructures were obtained with increasing plant extract concentration in the presence of Cu^2+^ ions in the solution supporting the formation of nanostructures. These nanoparticles were achieved with particle sizes of ~100 ± 30 nm (AgNPs) and ~300 ± 30 nm (AgNDs). Results demonstrated that AgNDs were more effective in terms of peroxidase, catalytic degradation, and antibacterial activity. AgNDs showed >90% catalytic degradation of methyl orange and methylene blue dyes. Additionally, AgNDs exhibited higher inhibition against *E. coli* than against *S. aureus*, suggesting added value in biomedical applications. Certain nanostructured forms, such as dendritic silver nanoparticles, can be applied to various fields in the future through green synthesis strategies.

## Data Availability

Data will be available upon request.
